# The role of PGE_2_ EP_4_ receptors in the regulation of endothelial barrier function

**DOI:** 10.1186/2050-6511-13-S1-A20

**Published:** 2012-09-17

**Authors:** Nora Kampitsch, Viktória Kónya, Rufina Schuligoi, Ákos Heinemann

**Affiliations:** 1 Institute of Experimental and Clinical Pharmacology, Medical University of Graz, 8010 Graz Austria

## Background

Prostaglandin E_2_ plays a crucial role in inflammation, including pain, fever and tumorigenesis. Inflammatory cells, fibroblasts and epithelium are the main source of PGE_2_ throughout an immune response. There are four known receptors for PGE_2_, E type prostanoid receptors 1–4 (EP_1,2,3,4_). According to recent reports, the EP_4_ receptor seems to be a potential target of therapeutic treatment for attenuating inflammation as well as increased endothelial permeability.

## Methods

Primary human microvascular endothelial cells of the lung (HMVEC-L) were cultured and transfected using siRNA approach. The mRNA level of the EP_4_ receptor was determined using RT-PCR. EP_4_ receptor protein expression was evaluated via Western blotting and flow cytometry. Changes in electrical impedance were measured using an ECIS application. Morphological alterations were observed via immunofluorescent staining of β-catenin and F-actin. Cell cycle and apoptosis analysis were performed using flow cytometry.

## Results

The pulmonary microvascular endothelial cells express the PGE_2_ receptor EP_4_, which was shown by flow cytometry and Western blotting. In endothelial cells, EP_4_ receptor protein expression was down-regulated to less than 40% by using the siRNA transfection approach. Also, the mRNA level of the EP_4_ receptor was significantly down-regulated below 15% using siRNA transfection. In the endothelial impedance measurements, the EP_4_ agonist and PGE_2_-induced barrier enhancement was significantly suppressed in EP_4_ receptor-silenced cells. Morphological studies revealed that the thrombin-induced disruption of endothelial monolayers could be reversed by stimulation of EP_4_ receptors. Cell-cell contacts were enhanced and stress fibre formation was prevented by pre-treatment with PGE_2_ and an EP_4_-selective agonist. PGE_2_ and the EP_4_ agonist did not induce any changes in the endothelial cell cycle; however, EP_4_ receptor activation appeared to be protective against staurosporine-induced apoptosis. Apoptotic cells were determined in the sub-G_1_ phase of the cell cycle.

## Conclusions

These data suggest EP_4_ receptor agonists as potential therapeutic intervention for diseases with increased vascular permeability such as acute lung injury.

